# Patient characteristics and healthcare use for high-cost patients with musculoskeletal disorders in Norway: a cohort study

**DOI:** 10.1186/s12913-024-12051-3

**Published:** 2024-12-18

**Authors:** Olav Amundsen, Tron Anders Moger, Jon Helgheim Holte, Silje Bjørnsen Haavaag, Line Kildal Bragstad, Ragnhild Hellesø, Trond Tjerbo, Nina Køpke Vøllestad

**Affiliations:** 1 Department for Interdisciplinary Health Sciences, Institute of Health and Society, University of Oslo, Oslo, Norway; 2 Department of Health Management and Health Economics, Institute of Health and Society, University of Oslo, Oslo, Norway; 3 Department of Public Health Science, Institute of Health and Society, University of Oslo, Oslo, Norway

**Keywords:** Healthcare utilisation, Musculoskeletal, Register-based research

## Abstract

**Background:**

A high proportion of healthcare costs can be attributed to musculoskeletal disorders (MSDs). A small proportion of patients account for most of the costs, and there is increasing focus on addressing service overuse and high costs. We aimed to estimate healthcare use contributing to high costs over a five-year period at the individual level and to examine if healthcare use for high-cost patients is in accordance with guidelines and recommendations. These findings contribute to the understanding of healthcare use for high-cost patients and help in planning future MSD-care.

**Methods:**

This study combined Norwegian registries on healthcare use, diagnoses, demographic, and socioeconomic factors. Patients (≥ 18 years) were included by their first MSD-contact in 2013–2015. We analysed healthcare use during the subsequent five years. Descriptive statistics were used to compare high-cost (≥ 95th percentile) and non-high-cost patients. Total healthcare contacts and costs for high-cost patients were examined stratified by number of hospitalisations and surgical treatments. Healthcare use of General Practitioners (GPs), physiotherapy, chiropractor and Physical Medicine and Rehabilitation physicians prior to the first hospitalisation or surgical treatment for a non-traumatic MSD was registered.

**Results:**

High-cost patients were responsible for 61% of all costs. Ninety-four percent of their costs were related to hospital treatment. Ninety-nine percent of high-cost patients had at least one hospitalisation or surgical procedure. Out of the high-cost patients, 44% had one registered hospitalisation or surgical procedure, 52% had two to four and 4% had five or more. Approximately 30–50% of patients had seen any healthcare personnel delivering conservative treatment other than GPs the year prior to their first hospitalisation/surgical treatment for a non-traumatic MSD.

**Conclusion:**

Most healthcare costs were concentrated among a small proportion of patients. In contrast to guidelines and recommendations, less than half had been to a healthcare service focused on conservative management prior to their first hospitalisation or surgical treatment for a non-traumatic MSD. This could indicate that there is room for improvement in management of patients before hospitalisation and surgical treatment, and that ensuring sufficient capacity for conservative care and rehabilitation can be beneficial for reducing overall costs.

**Supplementary Information:**

The online version contains supplementary material available at 10.1186/s12913-024-12051-3.

## Introduction

Musculoskeletal disorders (MSDs) include a wide range of conditions. These conditions can range from short-lived and mildly bothersome to chronic conditions that result in life-long disability, activity-limitations and reduced work and social participation [1, 2]. MSDs are one of the leading causes of years lived with disability in most countries according to the Global Burden of Disease Study [3]. Approximately one-third of all European workers are affected by MSDs, making it the most common work-related health problem in Europe [4]. The prevalence of MSDs increases with age, and it is expected that the societal burden associated with MSDs will increase significantly in the future [5, 6].

MSDs exert a substantial strain on societal economic resources. They represent one of the diagnostic groups with the highest direct healthcare costs and account for a significant proportion of countries’ healthcare budgets [7–10]. There are large variations in healthcare use for MSDs, where most people with MSDs do not seek any care, and a small proportion are responsible for the majority of number of healthcare contacts and costs [11–15]. Modern healthcare systems have an increased focus on reducing unwarranted costs and addressing overuse, and an overarching principle for cost-reduction is that the patient is treated at the lowest effective level of care [16–21]. The highest costs for MSD-care are associated with hospitalisation and surgical treatment, and guidelines recommend that most patients with MSDs should be managed in primary care and offered conservative treatment prior to surgical management. Yet, previous research indicates that many patients are treated surgically without having trialled appropriate conservative care [10, 18, 22–34].

Traumatic MSDs are routinely examined and treated in specialist care, where this management can be considered absolutely warranted [35]. Many non-traumatic MSDs, however, can be handled effectively in primary care with interventions such as self-management advice, exercise therapy, psychosocial interventions, and simple analgesics [31, 36]. Recommendations for conservative treatment for MSDs include using patient-centred care considering individual values, goals, and psychosocial factors, providing education and information, and include management that addresses physical activity and exercise [31, 37]. Stepwise care models in which high-quality conservative management and rehabilitation are attempted prior to specialist care referral have been suggested to increase the quality of care and reduce the costs of MSDs [38–42].

The first aim of this study was to estimate differences in healthcare contacts, costs, and patient characteristics between high-cost and non-high-cost patients. Secondly, we aimed to estimate how use of primary and specialist care services contributes to costs for high-cost patients. Thirdly, we aimed to investigate whether healthcare use for these patients is in concert with current recommendations and guidelines. The findings from this study can provide information on how different service use contributes to high costs in the long-term, how primary and specialist care are used in combination and how healthcare use for high-cost patients follow current recommendations and guidelines. These findings can contribute to the understanding of the characteristics and service use patterns of high-cost patients and aid in planning of future MSD-care.

## Methods

### Design and setting

This cohort study was conducted as part of the INnovations in use Of REGistry data (INOREG) project at the Institute of Health and Society, University of Oslo. The project combines several registries from 2008 to 2020 to create a cohort with healthcare use, costs, demographic and socioeconomic factors and outcomes for chronic diseases in Norway. The present study uses national registries with primary and specialist healthcare data, capturing all use of public healthcare in Norway. The reporting of this study adheres to the Strengthening the Reporting of Observational Studies in Epidemiology (STROBE) checklist for observational studies with the REporting of studies Conducted using Observational Routinely-collected Data (RECORD) extension [43, 44].

We used data on healthcare utilisation for MSDs from the Norwegian public healthcare service. Norway has a national universal healthcare system with the overarching goal of ensuring equal access to services for all residents [45]. The central government is responsible for specialist care services through regional health trusts, while primary care services is organized by the municipalities. All residents are members of the National Insurance Scheme, which allow access to health services, social benefits and pensions. Public funding is responsible for 85% of the total healthcare expenditure, which is the highest proportion of public funding in the WHO European Region [45]. The main providers for MSD-care in Norway include general practitioners (GPs), hospital services, physiotherapists and chiropractors. Additionally, contract specialists, municipal rehabilitation (mostly focused on elderly care with home-based rehabilitation and short-term rehabilitation stays in municipal institutions), municipal emergency care and specialist rehabilitation institutions are also involved. Other professionals such as naprapaths and osteopaths are also involved but work fully in the private market. Norway has had a Regular GP scheme in which all inhabitants are assigned their own GP since 2001 [45]. Ninety-six percent of the population are registered with a GP [46]. GPs function as gatekeepers in the Norwegian healthcare system and are responsible for coordinating care and referrals to specialist care [45]. Additionally, since 2008, physiotherapists with a master’s degree in treatment of MSDs (manual therapists) and chiropractors have had the right to refer to specialist healthcare and radiological examinations and to prescribe sick leave for up to 12 weeks for MSDs. GPs and physiotherapists are part of municipal primary care and receive capitation from the municipal for running the services.

The costs for consultations for GPs and physiotherapists are covered through a combination of fee-for-service reimbursement from The Norwegian Health Economics Administration (HELFO) and out-of-pocket payment from the patients [45]. Additionally, GPs and physiotherapists receive capitation from the municipality for running the service. Chiropractors operate as private practitioners and can set their own price but also receive a modest fee-for-service reimbursement from HELFO covering approximately 10% of the total cost [47]. Hospital care is provided by trusts owned by regional health authorities. Inpatient care is fully covered for patients, while outpatient care has an out-of-pocket cost similarly to that of GPs and physiotherapy services. Hospitals recieve approximately half of their income block grants from the state, while the other half are based on activity-based funding. A cost-sharing ceiling with a maximum limit for out-of-pocket costs exists to protect patients with high healthcare use from high costs.

### Sample selection

This study included all patients in Norway with an MSD-related healthcare contact in The Control and reimbursement of healthcare claims (KUHR) database, ICPC-2 Chapter L, or the Norwegian Patient Registry (NPR), ICD-10 Chapter M, during the years 2013–2015 and no MSD-related contacts during the preceding three years. Inclusion started in 2013 to ensure that the data from all healthcare services were complete. The time of the first MSD-related contact during 2013–2015 served as an index date, irrespective of which healthcare professional registered the first contact. Prior studies have defined a three-year wash-out period as optimal for excluding ongoing disorders and identifying new cases for common MSDs [48, 49]. The data on GP-services are complete from 2008, enabling us to use three years prior to the first contact as a washout-period. Patients younger than 18 years, or who died within five years after the index contact were excluded. Additionally, patients with MSD-diagnoses related to infection, malignancy, or inflammatory rheumatic diseases (ICPC-2 codes: L70, L71, L88 or L97. ICD-10 codes (M00-M03, M05-M08, M10, M11, M13, M30-M36, M45, M46, M60, M65 and M71 and M86) were excluded as the present study focused on symptom-based diagnoses. We used a follow-up period of five years for each patient, starting from the individual index date. To account for the COVID-19 pandemic, where it is expected that the lockdown had major impact on healthcare delivery, we excluded patients for whom the five-year follow-up extended beyond 12th March 2020.

### Data sources and variables

The selected registries provide a complete overview of public healthcare use, meaning that there was no loss to follow-up due to a lack of reporting. The fee-for-service reimbursements from HELFO are registered in KUHR, which allows the identification of contacts and costs for healthcare use at an individual level. Hospitals operate with activity-based funding based on the Nordic Diagnosis-Related Group (DRG) system to classify patients. This is registered in the NPR with a cost-weight, making it possible to calculate costs for contacts and procedures for each healthcare contact in the hospital.

We used data from the KUHR to capture primary healthcare utilisation. This allowed the calculation of use and costs for services provided by GPs, physiotherapists, chiropractors, contract specialists and municipal emergency care services. The NPR was used to capture specialist healthcare use and to calculate the corresponding costs. Statistics Norway and FD-Trygd were used for demographic and socioeconomic factors. The Norwegian Cause of Death Registry was used to exclude patients who died during the follow-up period.

All costs in this study are based on the reimbursement costs and activity-based funding registered in KUHR and NPR. This means that our data represents the cost for the public healthcare system to reimburse the actual services provided, but not the expenses of running the services or the patients’ out-of-pocket costs. The costs in this study includes the reimbursement costs for the services provided and do not include capitation from the municipality, the block grants to hospitals or the out-of-pocket costs for the patients. The true total cost is therefore greater than what we present in our findings, and there is an underestimation of the true costs for services that are partly funded by out-of-pocket costs. Hospitalisations and surgical treatment are funded fully through activity-based funding, meaning that our data includes the full costs for these services. Healthcare use related to contacts with GP, physiotherapy, chiropractor and outpatient contacts in hospital are partly funded by reimbursement from HELFO and partly by out-of-pocket expenses from the patient, meaning that the true cost for these services is higher than what we report in this study. The total costs related to treatment by chiropractors are also largely underestimated in our results, as this service is mainly financed by out-of-pocket costs.

A detailed list of the data sources, variables and their definitions used in this study are presented in Table 1. Categorizations and definitions are based on definitions by Statistics Norway if available or defined by the authors.

**Table 1:** Data sources, variables and variable definitions

Source	Variable	Definition
KUHR	Index date	First registered date with an MSD-related healthcare contact, if first date were registered in KUHR
	Diagnoses	International Classification of Primary Care, 2nd edition (ICPC-2) codes within chapter “L; Musculoskeletal” was used to identify contacts and costs related to MSDs. Patients registered with the specific codes L70 (Infection of musculoskeletal system), L71 (Malignant neoplasm musculoskeletal), L88 (Rheumatoid/seropositive arthritis and L97 (Neoplasm musculoskeletal benign/unspecific) were excluded
	Comorbidity	A previously adapted comorbidity index from GP-diagnoses based on ICPC-2 diagnoses, which have been validated to be used as an adjustment variable in epidemiological research in primary care databases [50]. This is a comorbidity index based on primary care data with eighteen selected diagnoses and the individual patient are assigned an index score based on number of diagnoses that can be identified. The comorbidity index was dichotomised into 0–1/2 or more
	Healthcare contact	Frequency of contacts for GP, physiotherapy, chiropractors, municipal emergency care and contract specialists with an MSD-diagnosis. Contact was defined as a healthcare contact with a fee indicating a face-to-face/video-consultation individually or group based and does not include fees that indicate simple communication, prescription writing or administrative work. This approach has shown high validity for the GP-service [51]. Included fees: GP: 2ad, 2ak, 2ae, 2ed Physiotherapy: A2a-f, A3a-b, A8a, A9a, A1a, A1d, c34 Chiropractors: K1, K2
	Healthcare cost	All reimbursement fees for contacts with an MSD-diagnosis. Costs are calculated in Norwegian currency but written in text as Euro (1€ = 11.3 NOK, per 19.02.2024)
	Geographical location	The municipality where the first GP-contact with an MSD-diagnosis were registered. The municipalities were classified as: “Large city, large municipality, small municipality” based on a national classification system from Statistics Norway [52]
NPR	Index date	First registered date with an MSD-related healthcare contact, if first date were registered in NPR
	Diagnoses	ICD-10 codes [53] within chapter “Diseases of the musculoskeletal system and connective tissue” were used to categorise contacts and costs related to MSDs. Patients registered with the specific codes related to infections (M00, M01, M02), malignant disease (M86) and inflammatory rheumatic disease (M05-M08, M10, M11, M13, M30-M36, M45, M46) were excluded. Musculoskeletal conditions include all ICD-10 codes chapter M, excluding those specified above, and the code G55. Injuries to the musculoskeletal system includes codes within chapter “Injury, poisoning and certain other consequences of external causes” related to MSD, codes S32-34, S40-S99 and T08-T13, excluding codes related to superficial injuries and wounds (Sx0 and Sx1)
	Healthcare contacts	Frequency of contacts after index date registered as outpatient contact or inpatient stay with an MSD-diagnosis. This includes contact with all healthcare personnel in specialist care
	Healthcare cost related to DRGs	Cost weight of DRG (corrected version) per MSD-related contact multiplied with cost of 1 DRG for the specific year. Cost of 1 DRG increased from 39,447 NOK in 2013 to 45,808 NOK in 2020. Costs are calculated in Norwegian currency but written in text as Euro (1€ = 11.5 NOK, per 09.07.2024)
	Procedure codes	Procedure codes using the Norwegian Clinical Procedure Codes for surgical and medical procedures [54]. Used to identify procedures for the first specialist care hospitalisation or surgical treatment for non-traumatic MSDs for high-cost patients
	Episode's discipline	Used to assess use of Physical Medicine and Rehabilitation physicians prior to high-cost patients first hospitalisation or surgical treatment for a non-traumatic MSD
	First hospitalisation or surgical treatment	First registered contact after index date indicating a hospitalisation or surgical treatment. Hospitalisation defined by code indicating overnight stay in hospital or out-date minimum one day after in-date. Surgical treatment defined by the Norwegian Clinical Procedure Codes for surgical procedures and diagnosis indicating a surgical treatment for a non-traumatic MSD [54]
Statistics Norway	Age	Age in years at index date
	Gender	Male/female
	Education	Highest registered education. Categorised: 13 years or less/more than 13 years
	Income	Income registered the year before inclusion. Categorised as below/above median
	Immigrant background	Categorised: No immigration background or Any immigration background (Includes: first generation immigrant, Norwegian-born second-generation immigrant, one foreign parent, born outside Norway from Norwegian parents)
FD-Trygd	Disability pension	If the patient is registered on disability pension prior to inclusion
Norwegian Cause of Death Registry	Date of death	Date of death were used to exclude patients that died during the five-year follow-up period

*Abbreviations*: *KUHR* The Control and Reimbursement of Healthcare Claims, *NPR* Norwegian Patient Registry, *MSD* Musculoskeletal disorder, *GP* General practitioner, *ICPC-2* International Classification of Primary Care, 2nd version, *ICD-10* International Statistical Classification of Diseases and Related Health Problems 10th Revision, *DRG* Nordic Diagnosis-Related group

### Statistical analysis

High-cost users were defined as individuals whose cost during the five-year period was equal to or exceeded the 95th percentile. There is no consensus on the definition of high-cost patients, and the definition varies among studies [14, 55–57]. In our data, the 95th percentile for total costs in the five year-period was 6 010 Euro (€), for comparison of high-cost and non-high-cost patients.

We examined the number of hospitalisations and surgical treatments for the high-cost patients, and the distribution of number of total healthcare contacts and total healthcare costs for patients with none, one, two to four, or five or more hospitalisations or surgical treatments for MSDs during the follow-up period. These findings were illustrated with bar graphs.

For patients with a hospitalisation or surgical treatment for a non-traumatic MSD, we examined whether the patient had seen healthcare personnel delivering conservative care prior to the first hospitalisation/surgical treatment. Conservative treatment and rehabilitation are recommended as first-line treatments for conditions such as osteoarthritis [33, 52, 58], spinal disorders [18, 25, 32], shoulder pain [28, 30, 59, 60], and knee disorders [61–63]. We categorised patients based on the diagnosis for the hospitalisation or surgical treatment, using the categories: Osteoarthritis, spinal disorders, shoulder pain and knee disorders. The main providers of conservative care in Norway are GPs, physiotherapists, chiropractors and Physical Medicine and Rehabilitation (PM & R) physicians in specialist care. We assessed whether the patients had registered a contact with any of these healthcare services prior to their first hospitalisation or surgical treatment for a non-traumatic MSD. We included contacts with GPs, physiotherapists, chiropractors and PM & R physicians the last year prior to the hospitalisation/surgical treatment to ensure that the contact was relatively close to the specialist care contact. Due to the uncertainty of the specific diagnosis codes used in primary care, we did not restrict the exact diagnosis but included all contacts with any MSDs. There is no standardized method for identifying which healthcare contacts are relevant to the condition the patient were treated in specialist care for, and which contacts are related to other musculoskeletal problems. It could be argued that our definition is too strict, as it is possible that conservative treatment more than one year prior to the specialist care contact may be relevant, or not strict enough, as it does not restrict on specific diagnosis codes relevant to the specialist care contact. To account for this, we included analyses with both a stricter and a less strict definition as a sensitivity analysis. The less strict definition included all contacts for any MSDs (including contacts where fees indicated simple communication, prescription writing or administrative work) between the index date and the specialist care contact. The stricter definition included only contacts the year before which included a diagnosis code relevant for the hospitalisation/surgical treatment.

## Results

**Fig. 1:**
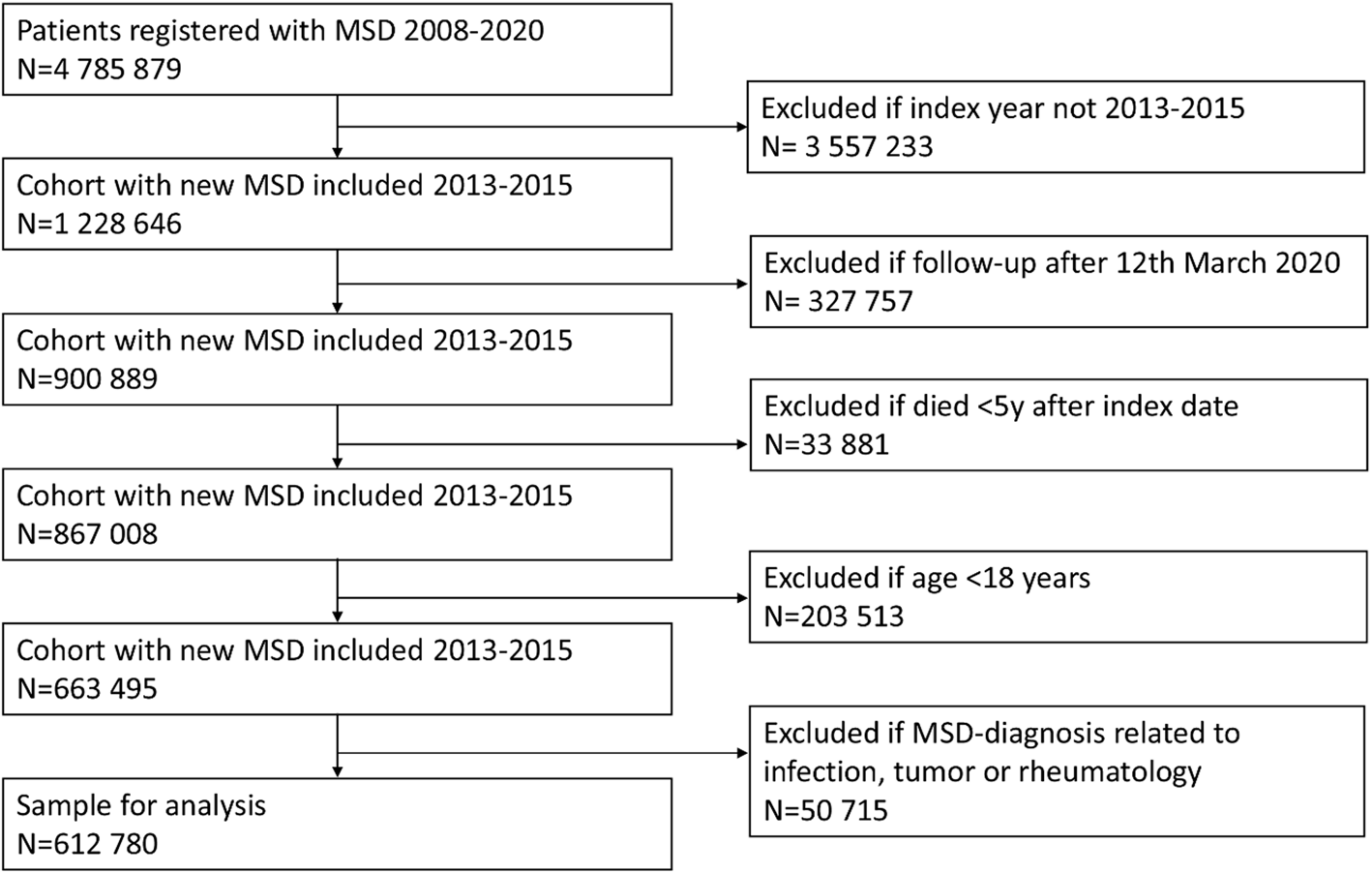
Flowchart of the sample selection process. MSD = Musculoskeletal disorder

The initial database included 4 785 879 patients who were registered with an MSD-diagnosis between 2008 and 2020. We excluded patients based on index year, follow-up time after the COVID-19 lockdown, age less than 18 years, specific diagnosis and death during the five-year period. This led to a final sample consisting of 612 780 patients (Fig. 1).

Patients with healthcare costs above the 95th percentile (*N* = 30 639) were older, had less education, were more likely to receive disability pensions prior to the index date, fewer patients with immigrant background, more comorbidity than non-high-cost patients (*N* = 582 141) (Table 2).

**Table 2:** Patient characteristics for high-cost and non-high-cost patients. *N* = 612 780

	High-cost patients *N* = 30 639 (5%)	Non-high-cost patients *N* = 582 141 (95%)	*P*-value*
Age, years (mean (SD))	58 (18)	44 (17)	< .001
Gender, female	50.9%	48.3%	< .001
Education, more than 13 years	28.7%	39.5%	< .001
Income in € (mean (SD))^+^	35 011 (21 011)	36 435 (22 256)	< .001
Disability pension before index	5.0%	3.5%	< .001
Immigrant background	14.1%	22.7%	< .001
Comorbidity, 2 or more^a^	6.4%	2.6%	< .001

^*^T-test/chi-square ^+^Removed patients registered with negative income (0.01%) and income above 99th percentile ^a^Comorbidity index based on the International Classification of Primary Care (ICPC-2) [50]

**Fig. 2:**
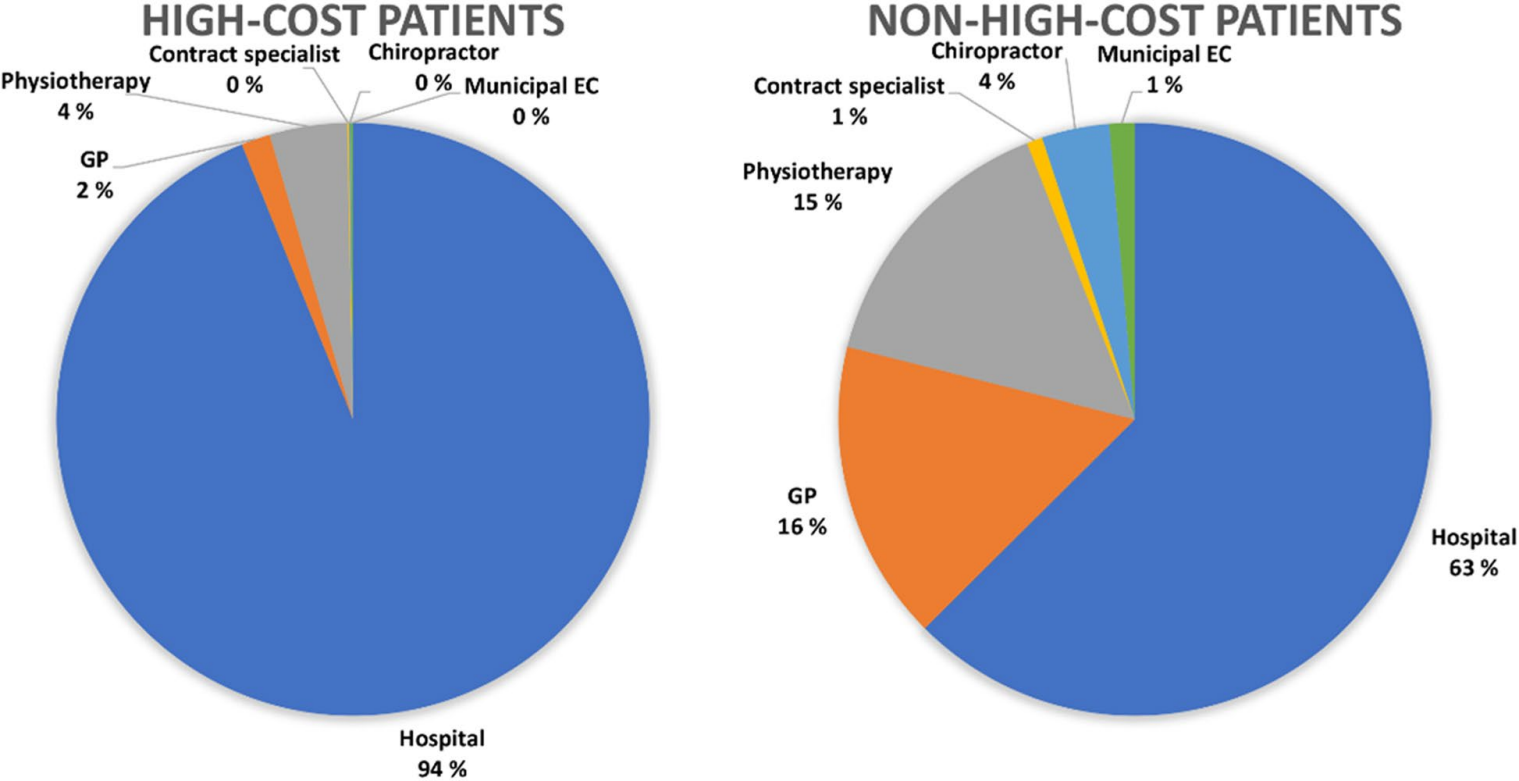
Pie diagram showing the relative proportion of healthcare costs for each healthcare service, for high-cost patients and non-high-cost patients. GP = general practitioner. EC = emergency care

High-cost patients had a median cost of €10 717 (IQR 6 243), while non-high-cost patients had a median cost of €102 (IQR 267). High-cost patients had a median of 23 (IQR 40) healthcare contacts in the five-year follow-up period, while non-high-cost patients had 5 (IQR 10) healthcare contacts. High-cost patients accounted for 61% of all costs during the follow-up period. Hospital costs accounted for 94% of all costs for the high-cost patients, and 63% of all costs for non-high-cost patients (Fig. 2). The cost distributions per service for high-cost patients and non-high-cost patients are illustrated in Fig. 2.

**Fig. 3:**
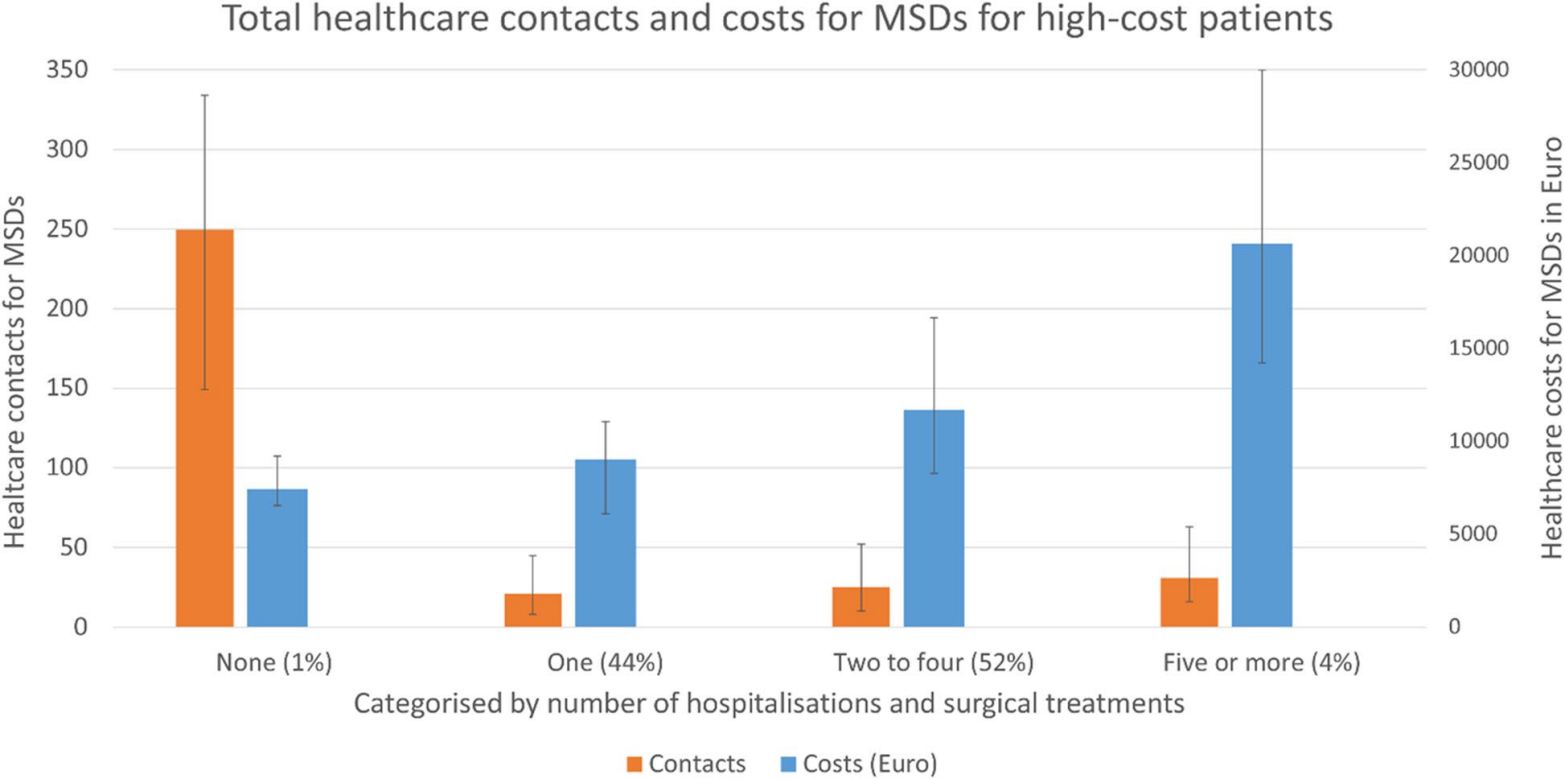
Bar graphs showing total healthcare contacts and costs (median and interquartile range) for high-cost patients (*N* = 30 639) during the five-year follow-up period, categorised by number of hospitalisations and surgical treatments

Ninety-nine percent of high-cost patients had at least one hospitalisation or surgical procedure. Out of the high-cost patients, 44% had one registered hospitalisation or surgical procedure, 52% had two to four hospitalisations or surgical procedure and 4% had five or more. Figure 3 shows the median total healthcare contacts and costs for these categories during the five-year follow-up period. The patients with no registered hospitalisation or surgical procedure had a median of 249.5 healthcare contacts over the five-year period, and 86% of these were registered with a physiotherapist. The total healthcare costs increased significantly based on the number of hospitalisations or surgical treatments.

When assessing healthcare use prior to hospitalisation or surgical treatment, we only included patients with non-traumatic MSDs, where guidelines suggest that conservative treatment and rehabilitation should be trialled as a first-line treatment. Fifty-three percent of the high-cost patients (*N* = 16 204) had at least one hospitalisation or surgical treatment for a non-traumatic MSD during the follow-up period. When identifying the first hospitalisation or surgical treatment during the follow-up period for these patients, 46% was related to osteoarthritis, 28% to spinal disorders, 5% to shoulder disorders, 5% knee disorders and 16% to other non-traumatic MSDs. All specific ICD-10 diagnoses included in each diagnostic category (Supplementary 1) and the most commonly registered procedures for each diagnostic category (Supplementary 2) are included in the supplementary material.

Eighty percent of patients who had a hospitalisation or surgical treatment for osteoarthritis, spinal, shoulder- or knee disorders, or other non-traumatic MSDs, had seen their GP the year before (Table 3). Approximately 30–50% of patients with contacts related to osteoarthritis, and spinal, shoulder and knee disorders had seen any healthcare personnel delivering conservative treatment other than GPs the year prior to their first hospitalisation or surgical treatment (Table 3). Depending on the definition, this varies from 16–35% with a stricter definition to 38–59% with a less strict definition (Supplementary 3).

**Table 3:** Healthcare use the year before first hospitalisation or surgical treatment for non-traumatic MSDs. Categorised by diagnosis registered for the first hospitalisation or surgical treatment. Only includes high-cost patients with at least one hospitalisation or surgical treatment for non-traumatic MSDs (*N* = 16 204)

	GP	Physiotherapy	Chiropractor	PM & R	Any other than GP
Diagnostic category	Proportion with one or more contacts	Proportion with one or more contacts	Proportion with one or more contacts	Proportion with one or more contacts	Proportion with one or more contacts
Osteoarthritis *N* = 7 509 (46%)	84.7%	34.3%	5.9%	4.7%	41.0%
Spinal disorders *N* = 4 538 (28%)	82.8%	21.1%	20.6%	6.7%	41.4%
Shoulder *N* = 755 (5%)	86.9%	41.7%	8.2%	4.9%	49.9%
Knee *N* = 821 (5%)	78.9%	27.4%	6.2%	1.2%	32.0%
Other MSDs *N* = 2 581 (16%)	63.3%	18.4%	6.5%	2.1%	24.7%
Total *N* = 16 204 (100%)	80.4%	28.1%	10.3%	4.7%	38.5%

*GP* General practitioner, *PM & R* Physical Medicine and Rehabilitation

## Discussion

### Main findings

Our study identified differences in patient characteristics between high-cost (5%) and non-high-cost patients (95%). High-cost patients were older, had less education, had more comorbidity and a higher proportion were on disability pensions than non-high-cost patients. There were a lower proportion of immigrants and more people living in small municipalities, and there was only small differences in gender. High-cost patients accounted for more than 60% of all costs in the 5-year period. Ninety-four percent of their costs were related to specialist healthcare. The findings showed that the total costs increased significantly based on the number of hospitalisations and surgical treatments a patient had during the five-year period. Only one percent of high-cost patients had no hospitalisations or surgical treatments. These patients had a healthcare use characterised by very many healthcare contacts, primarily related to physiotherapy use. More than half of the high-cost patients had a hospitalisation or surgical treatment for a non-traumatic MSD. Most of these patients had been to their GP, and 30–50% of these patients had contacts with healthcare personnel delivering conservative care other than GP before the first hospitalisation or surgical treatment.

### Comparison of high-cost and non-high-cost patients

Previous studies have shown that higher age, education, comorbidity and being on disability pension are associated with a greater burden of MSDs and poorer outcomes [64, 65]. Hence, our finding that being among the high-cost users is associated with these factors could therefore be explained by clinical factors not present in the data used in the current study. We found a greater proportion of immigrants in the non-high-cost group, suggesting that they are to a larger extent managed in primary care compared to native Norwegians [15]. Alternatively, the higher prevalence of MSD among immigrants in Norway may reflect a lower average disease burden [66]. It is also noteworthy that there were only small gender differences in the proportions of high-cost-groups.

### Healthcare use for high-cost patients

Previous research has shown that surgical procedures and hospitalisation account for the highest costs related to MSDs in the short-term [22], and our findings demonstrate that this is also the case in a five-year perspective. Our findings show that older patients are more likely to be high-cost users, suggesting that costs will increase significantly in the future as the population ages. Current models of care may not be sustainable for handling this increased burden [67, 68], highlighting the importance of researching resource use and discussing models of care that lead to prevention of disease burden and sustainable costs of MSD management.

Fifty-three percent of high-cost patients had a hospitalisation or surgical treatment for a non-traumatic MSD, such as osteoarthritis, spinal, shoulder and knee disorders. Conservative management and rehabilitation are generally recommended as first-line treatments for these conditions [18, 25, 30–33, 52, 58–63]. Most patients have seen their GP prior to their first hospitalisation or surgical treatment for a non-traumatic MSD. GPs represent an essential part of conservative care for MSDs, as this is the most common point of entry when seeking help from the healthcare system. GPs can provide effective care for MSDs with examinations, information, reassurance, advice and pharmacological treatment, as recommended first-line treatment for MSDs [31, 36]. Most patients that seek healthcare for MSDs only require help from their GP, and most people are managed with only one or a few contacts with their GP, suggesting that this is an effective management option for most patients [15].

For the patients that have a hospitalisation or surgical treatment for non-traumatic MSDs, such as osteoarthritis and spinal, shoulder and knee disorders, only 30–50% patients had seen healthcare personnel delivering conservative treatment, other than GPs, the year prior to the hospitalisation or surgical treatment. This finding is supported by previous research showing that 30–40% of patients receive appropriate nonsurgical care for osteoarthritis, 35% of patients receive a conservative plan of care the year prior to elective lumbar spine surgery and 65% of patients with rotator cuff disorders receive a nonsurgical management program [26–29]. This may suggest that although most people with MSDs have contact with their GP, it is a relatively low proportion of patients that try other conservative care and rehabilitation before they are treated in specialist care.

The finding that less than half of patients see healthcare personnel delivering conservative treatment other than GPs prior to their first hospitalisation or surgical treatment for a non-traumatic MSD appears to contrast with recommendations and guidelines. There are numerous reasons for why treatment may or may not adhere to clinical guidelines in each individual case, such as unfamiliarity with guidelines, disagreement with recommendations, pressure from patients and fear of repercussions [43–45]. Additionally, guidelines are only meant to support decision-making, and not intended as replacement for clinical judgement or to be followed meticulously [46, 47]. In each individual case, there could be important factors, e.g., specific clinical characteristics or unremitting high disability and pain intensity, that explain why treatment did or did not follow guidelines.

The lack of clinical data in our study makes it impossible to determine whether there are important clinical factors that explain why patients are referred directly to specialist care and are not treated in primary care. Patients with spinal pain referred to PM & R specialist care in Norway have been found to have more severe symptoms, poorer quality of life, less education and greater psychosocial distress than patients treated in primary care [69]. In contrast, a study on hand osteoarthritis in Norway showed that patients who were directly referred for surgical care had less pain and fewer limitations than patients who underwent rehabilitative interventions before referral [70]. Fewer than half of the patients in our study had seen healthcare personnel delivering conservative treatment other than GPs the year preceding their first hospitalisation or surgical treatment. This was seen even for diagnoses where guidelines recommend rehabilitation as a first line treatment. It is unlikely that specific clinical characteristics or symptom severity can explain why such a substantial proportion of patients have not undergone conservative management or rehabilitation.

### High-cost users in primary care

Our data show that one percent of high-cost patients had high costs related to long-term follow-up in primary care, most commonly due to many physiotherapy contacts. This finding is similar to a previous study from Denmark [71]. Patients with high levels of physiotherapy use have been found to have high pain and disability and to have negative health and illness perceptions, lower levels of internal health locus of control, poor self-management and high emotional distress [72–74]. Patients who frequently use physiotherapy report less improvement in clinical outcomes than patients with less use [74], and studies indicate that a greater number of physiotherapy contacts does not lead to improved clinical outcomes [75–77]. This makes it important to discuss whether the high use of physiotherapy contacts in our findings may indicate an overuse of physiotherapy. Combined with the finding that many patients with MSDs do not go receive recommended conservative care or rehabilitation prior to hospitalisation or surgical treatments, this may suggest that it could be beneficial with a shift towards less use per patient to free up space for more new patients in the physiotherapy service. On the other hand, healthcare delivery for patients with persistent MSDs and chronic conditions is complex and the value of providing healthcare may not be captured sufficiently by traditional clinical outcome measures [78]. The high use of primary care treatment could lead to reduced use of more expensive specialist care interventions, resulting in a net benefit. The complex interplay of overuse and underuse illustrates the difficulty of optimizing healthcare delivery for patients with MSDs, and the balance between resource savings and personalized patient care remains an important challenge for healthcare systems.

### Future perspectives – Room for improvement?

Initiatives to align care to guideline recommendations have shown promising results for reduction of unwarranted diagnostic imaging and specialist care use [18, 79–82]. The implementation of a structured model of care for hip and knee osteoarthritis has been shown to increase the quality of care and be cost-effective, primarily through reduced surgery rates [42]. Moreover, stepwise care approaches have been shown to be cost-effective for knee disorders [41]. Studies have also shown that exercise therapy and patient education can lead to reduced surgery rates [83–87]. Our findings indicate that the commonly recommended stepwise care model in which patients use conservative management and rehabilitation prior to more expensive specialist care treatment is underutilised in current practice. Increased use of conservative treatment such as patient education, rehabilitation, and exercise therapy as first-line treatment, as recommended in guidelines, could offer cost-savings by reducing or postponing more expensive specialist care procedures.

There is a low capacity for both primary and specialist care rehabilitation compared to the demand, resulting in long waiting times before patients can access rehabilitation in Norway [88]. Early access to physiotherapy is associated with lower MSD-related costs and lower use of imaging, injections and surgical interventions compared to delayed access, indicating that the timing of care is important [89]. Guidelines state that patients who have undergone surgical procedures are prioritized for primary care rehabilitation services, while patients with acute and chronic MSDs have lower priority [90]. A lack of capacity and reduced access to rehabilitation services before surgical procedures are likely to be important barriers for why stepwise care approaches are not routinely used in current practice.

To allow more patients to be provided appropriate conservative care and rehabilitation prior to specialist care treatment, it is paramount that patients can access these services. Our findings indicate that there may be an underuse of the step between GP-care and specialist care, where patients are provided further conservative care and rehabilitation in addition to the care provided by the GP. To achieve this, it most likely requires a shift within the services to focus on providing care for more patients. Our data suggests that some patients receive a very high number of physiotherapy contacts, and it may be possible that some of these resources could be allocated to allow more new patients to access the services. More importantly, it is important to acknowledge that this also most likely requires significant shifts in investments towards primary healthcare as the demand for healthcare services are higher than the current capacity.

### Strengths and limitations

This study provides novel contributions to the existing body of literature on high-cost users in musculoskeletal healthcare. Most prior studies have focused on costs related to services rather than individuals, used short timeframes, or focused on predictive models [14, 22, 47, 71, 91–94]. Our study utilised registry data and allowed us to use a longer timeframe for defining high-cost users compared to previous research. By using individualised data to assess healthcare use and costs, we provide detailed information on healthcare use for patients with high costs, and whether the healthcare use is in concordance with current recommendations and guidelines.

Using registries that include a large population linked at the individual level provides a unique dataset with complete information on public primary and specialist healthcare use and demographic and socioeconomic factors. This makes it possible to create a comprehensive cohort without problems with selection bias that influence the validity of the findings [95]. This ensures that the study provides a more accurate overview of real-life clinical practice than what is possible to achieve with other designs [96].

An important limitation of the study is the lack of clinical data. This makes it impossible to know if there are important clinical characteristics that lead to different management of patients in our study. For MSDs, clinical factors such as pain intensity, disability and interference with daily life and activity is a very important part of decision making for different management options for MSDs, and this information would likely contribute to a better understanding of healthcare use for patients [74, 97].

Our study uses primary care diagnoses and defined contacts from fee codes that indicate face-to-face, group or video consultation, an approach that has been shown to have high validity for the GP-service [51]. It is important to acknowledge that differing coding behaviour between clinicians and professions, and different software between clinics might challenge the validity of diagnosis codes used in registry data [98]. To account for this, we included all MSDs as one broad category rather than categorizing on more detailed codes, except for the most expensive specialist care contact, as this would most likely reduce accuracy.

Our results only capture the reimbursement costs for the public healthcare system. This means that our reported healthcare costs underestimate the true cost of healthcare use, as these services are partially financed from out-of-pocket expenses and receive additional funding for running the services. As our data are based on reimbursement costs, we most likely underestimate the cost of chiropractors, as patients with high use of chiropractors cannot reach the 95th percentile for costs due to low reimbursement rates for these services. Another limitation is that we can only include patients who use the public healthcare system. There is an ambition in Norway that all parts of the population should have equal access to public high-quality healthcare services [45] and public healthcare represents 86% of all healthcare use in 2022 [99]. The use of private healthcare increased significantly during the recent years, and in 2021, approximately 13% of the population had private health insurance [100]. This means that there is a substantial amount of healthcare use on which we have no information, as privatized healthcare is not included in the registries. Pain medications represent an important aspect of management of MSDs, and a significant part of healthcare costs. Unfortunately, we were not able to access The Norwegian Prescription Database within the projects timeframe and have no information on medication use.

Previous studies show that the challenges related to healthcare use for MSDs in discordance with guidelines are a global problem. Direct comparisons between countries are difficult due to large differences in healthcare organisations, welfare systems and social structures. We believe that the findings of our study are generalizable to other countries with similar healthcare organisations and structures, but that the challenges highlighted in this study are relevant also in a more global context.

## Conclusion

The five percent of patients with the highest healthcare costs were responsible for more than 60% of all costs for MSD-related care over a five-year period. High-cost patients were older, had less education, had more comorbidities and were more likely to receive disability pensions than non-high-cost patients were. Total costs increased significantly based on the number of hospitalisations or surgical treatments a patient had during the five-year period. We found a relatively low use of healthcare services delivering conservative care and rehabilitation, other than GPs, the year before patients’ first hospitalisation or surgical treatment for non-traumatic MSD. This contrasts with guidelines where conservative management and rehabilitation are recommend before specialist referral. Previous studies have shown that stepwise care approaches and interventions such as patient education and exercise therapy can reduce surgery rates and costs. Our findings indicate that there may be an underuse of the step between GP-care and specialist care, and that it could be beneficial to focus on offering patients with non-traumatic MSDs the opportunity to try further conservative management and rehabilitation before being referred to specialist care management. It is important to acknowledge that this most likely requires significant shifts in prioritisations and investments towards primary healthcare.

## Supplementary Information

Supplementary Material 1.

Supplementary Material 2.

Supplementary Material 3.

## Data Availability

The datasets used in the current study are based on national registries and are not publicly available. Access to pseudonymized data from the national registries is only granted through application to the Norwegian Centre for Research Data and Regional Committees for Medical and Health Research Ethics. Other data and materials can be obtained from the corresponding author upon reasonable request.

## Abbreviations

MSDs: Musculoskeletal disorders
INOREG: INnovations in use of REGistry data
STROBE: Strengthening the Reporting of Observational Studies in Epidemiology
RECORD: REporting of studies Conducted using Observational Routinely-collected Data
GP: General practitioner
HELFO: The Norwegian Health Economics Administration
KUHR: The Control and Reimbursement of Health Care Claims
NPR: Norwegian Patient Registry
FD-Trygd: Statistics Norway's Social Security Event History Database
ICPC-2: International Classification of Primary Care, 2nd version
ICD-10: International Classification of Diseases
DRG: Nordic Diagnosis-Related Group
PM & R: Physical Medicine and Rehabilitation
