# Supplementary material for: Patient characteristics and healthcare use for high-cost patients with musculoskeletal disorders in Norway: a cohort study
Source: BMC Health Serv Res. 2024 Dec 18;24:1583. doi: 10.1186/s12913-024-12051-3 (PMC11653887; doi:10.1186/s12913-024-12051-3)
Supplement: Supplementary file 1 — Supplementary Material 1. [file 12913_2024_12051_MOESM1_ESM.docx]

Supplementary 1: All diagnoses for the most expensive specialist care contact for the high-cost patients, N= 16 204

| Diagnoses |  |
| --- | --- |
| **Osteoarthritis** | **7 509 (100)** |
| M15 Polyarthrosis | 21 (0.3) |
| M16 Hip osteoarthritis | 4 658 (62.0) |
| M17 Knee osteoarthritis | 2 342 (31.2) |
| M18 Arthrosis of first carpometacarpal joint | 49 (0.7) |
| M19 Other arthrosis | 439 (5.9) |
| **Spinal pain** | **4 538 (100)** |
| M43 Other deforming dorsopathies | 145 (3.2) |
| M47 Spondylosis | 112 (2.5) |
| M48 Other spondylopathies | 1 263 (27.8) |
| M50 Cervical disc disorders | 595 (13.1) |
| M51 Other intervertebral disc disorders | 1 865 (41.1) |
| M53 Other dorsopathies, not elsewhere classified | 40 (0.9) |
| M54 Dorsalgia (back pain) | 514 (11.3) |
| G55 Nerve root and plexus compressions in diseases classified elsewhere | 4 (0.1) |
| **Knee** | **821 (100)** |
| M22 Disorders of patella | 37 (4.5) |
| M23 Internal derangement of knee | 784 (95.5) |
| **Shoulder** | **755 (100)** |
| M75 Shoulder lesions | 755 (100) |
| **Other** | **2 581 (100)** |
| M20 Acquired deformities of fingers and toes | 425 (16.5) |
| M21 Valgus deformity, not elsewhere classified | 51 (2.0) |
| M24 Other specific joint derangements | 284 (11.0) |
| M25 Other joint disorders, not elsewhere classified | 159 (6.2) |
| M40 Kyphosis and lordosis | 7 (0.3) |
| M41 Scoliosis | 27 (1.1) |
| M42 Spinal osteochondrosis | <5 |
| M49 Spondylopathies in diseases classified elsewhere | <5 |
| M61 Calcification and ossification of muscle | <5 |
| M62 Other disorders of muscle | 96 (3.7) |
| M66 Spontaneous rupture of synovium and tendon | 33 (1.3) |
| M67 Other disorders of synovium and tendon | 69 (2.7) |
| M70 Soft tissue disorders related to use, overuse and pressure | 80 (3.1) |
| M72 Fibroblastic disorders | 164 (6.4) |
| M76 Enthesopathies of lower limb, excluding foot | 45 (1.7) |
| M77 Other enthesopathies | 47 (1.8) |
| M79 Other soft tissue disorders, not elsewhere classified | 341 (13.2) |
| M80 Osteoporosis with pathological fracture | 149 (5.8) |
| M81 Osteoporosis without pathological fracture | 7 (0.3) |
| M84 Disorders of continuity of bone | 263 (10.2) |
| M85 Other disorders of bone density and structure | 14 (0.5) |
| M87 Osteonecrosis | 73 (2.8) |
| M89 Other disorders of bone | 73 (2.8) |
| M90 Osteopathies in diseases classified elsewhere | <5 |
| M91 Juvenile osteochondrosis of hip and pelvis | <5 |
| M92 Other juvenile osteochondrosis | 34 (1.3) |
| M93 Other osteochondropathies | 49 (1.9) |
| M94 Other disorders of cartilage | 9 (0.4) |
| M95 Other acquired deformities of musculoskeletal system and connective tissue | 28 (1.1) |
| M96 Postprocedural musculoskeletal disorders, not elsewhere classified | 39 (1.5) |
| M99 Biomechanical lesions, not elsewhere classified | 60 (2.3) |
