# Supplementary material for: Patient characteristics and healthcare use for high-cost patients with musculoskeletal disorders in Norway: a cohort study
Source: BMC Health Serv Res. 2024 Dec 18;24:1583. doi: 10.1186/s12913-024-12051-3 (PMC11653887; doi:10.1186/s12913-024-12051-3)
Supplement: Supplementary file 2 — Supplementary Material 2. [file 12913_2024_12051_MOESM2_ESM.docx]

Supplementary 2: Procedures registered in the most expensive specialist care contact for high-cost patients.

| Diagnoses | N | Most common procedures |
| --- | --- | --- |
| Osteoarthritis | 7 509 | Joint prothesis (88%)  Arthrodesis (2%)  Resection bone (1%)  Arthroscopy (1%)  Other (8%) |
| Spinal disorders | 4 538 | Microdiscectomy/discectomy (48%)  Decompression (31%)  Spinal fusion (3%)  Not registered (11%)  Other (7%) |
| Knee | 821 | Meniscus surgery (56%)  Arthroscopy (17%)  Joint capsule/ligament surgery (16%)  Surgery for habitual dislocation (1%)  Resection bone (1%)  Other (9%) |
| Shoulder | 755 | Resection bone (36%)  Arthroscopy (24%)  Tendon/muscle surgery (23%)  Joint capsule/ligament surgery (8%)  Surgery for habitual dislocation (3%)  Not registered (2%)  Other (4%) |
| Other MSDs | 2 581 | Surgery ankle/foot/toe (27%)  Surgery hip/thigh (11%)  Surgery knee (9%)  Surgery shoulder (8%)  Surgery wrist/hand (8%)  Surgery spine (3%)  Not registered (19%)  Other (15%) |
| Total | 16 204 |  |

**Procedures based on NCSP_x codes from the Norwegian Clinical Procedure Codes for surgical and medical procedures:**

Joint prothesis: N(X)B00-99

Tendon/muscle surgery: N(X)L00-99

Joint capusule/ligament N(X)E00-99

Resection bone: N(X)K10-19

Arthrodesis: N(X)D30-59

Microdiscectomy/discectomy: ABC07-26

Decompression: ABC30-99

Spinal fusion: NAG30-99

Meniscus surgery: NGD00-99

Arhtroscopy: N(X)A11

Surgery for habitual dislocation: N(X)H72

Surgery ankle/foot/toe: NH(Xxx)

Surgery shoulder: NB(Xxx)

Surgery wrist/hand: ND(Xxx)

Surgery hip/thigh: NF(Xxx)

Surgery knee: NG(Xxx)

Surgery spine: NC(Xxx)

Surgery elbow/arm: NC(Xxx)
