# Supplementary material for: Patient characteristics and healthcare use for high-cost patients with musculoskeletal disorders in Norway: a cohort study
Source: BMC Health Serv Res. 2024 Dec 18;24:1583. doi: 10.1186/s12913-024-12051-3 (PMC11653887; doi:10.1186/s12913-024-12051-3)
Supplement: Supplementary file 3 — Supplementary Material 3. [file 12913_2024_12051_MOESM3_ESM.docx]

Supplementary 3: Sensitivity analysis

**Table 1: Percentage with at least one MSD-contact for each service before their first hospitalisation/surgical treatment, categorised by diagnosis registered on the most expensive contact. For osteoarthritis, spinal-, shoulder- and knee disorders only included contacts with diagnosis specific to the specialist care procedure, less than one year before procedure.**

|  | GP | Physiotherapy | Chiropractor | PM & R | Any other than GP |
| --- | --- | --- | --- | --- | --- |
| **Diagnosis** | Proportion with one or more contacts | Proportion with one or more contacts | Proportion with one or more contacts | Proportion with one or more contacts | Proportion with one or more contacts |
| Osteoarthritis | 63.8% | 24.8% | 0.0% | 4.1% | 28.0% |
| Spinal | 75.1% | 16.1% | 19.1% | 6.1% | 35.8% |
| Shoulder | 73.6% | 30.0% | 2.9% | 3.6% | 35.0% |
| Knee | 63.6% | 15.2% | 1.3% | 0.0% | 16.1% |

Osteoarhtitis: Only included contacts with ICPC-2 codes L89-91 and ICD-10 codes M15-M19.
Spinal disorders: Only included contacts with ICPC-2 codes L01-03, L83-86, and ICD-10 codes M47-48, M50-51 and M54.
Shoulder: Only included contacts with ICPC-2 codes L08, L92, and ICD-10 codes M75.
Knee: Only included contacts with ICPC-2 codes L15, L96, and ICD-10 codes M22 and M23.

**Table 2: Percentage with at least one MSD-contact for each service before their first hospitalisation/surgical treatment, categorised by diagnosis registered on the most expensive contact. All contacts with any MSDs at any time between index date and specialist care procedure.**

|  | GP | Physiotherapy | Chiropractor | PM & R | Any other than GP |
| --- | --- | --- | --- | --- | --- |
| **Diagnosis** | Proportion with one or more contacts | Proportion with one or more contacts | Proportion with one or more contacts | Proportion with one or more contacts | Proportion with one or more contacts |
| Osteoarthritis | 92.3% | 44.9% | 11.8% | 5.6% | 53.3% |
| Spinal | 87.9% | 28.4% | 28.3% | 9.0% | 51.7% |
| Shoulder | 90.7% | 50.5% | 16.3% | 7.3% | 59.3% |
| Knee | 84.9% | 33.6% | 9.9% | 2.6% | 40.4% |
| Other MSDs | 77.8% | 28.5% | 12.3% | 3.6% | 37.6% |
| Total | 88.3% | 37.4% | 16.6% | 6.2% | 50.0% |
